# Single-Stranded Variable Fragment Gene Libraries Built for Phage Display: An Updated Review of Design, Selection and Application

**DOI:** 10.4014/jmb.2407.07049

**Published:** 2024-10-24

**Authors:** Caio Coutinho de Souza, Juliane Corrêa Glória, Eliza Raquel Duarte da Silva, André de Lima Guerra Corado, Kelson Ávila Graça de Alcântara, Isabelle Bezerra Cordeiro, Edmar Vaz de Andrade, Luis André Morais Mariúba

**Affiliations:** 1 Programa de Pós-graduação em Biotecnologia (PPGBIOTEC), Universidade Federal do Amazonas (UFAM), Manaus, AM, Brazil; 2 Laboratório de Diagnóstico e Controle de Doenças Infecciosas na Amazônia (DCDIA), Instituto Leônidas e Maria Deane (ILMD/Fiocruz-Amazônia), Manaus, AM, Brazil; 3 Programa de Pós-Graduação em Biologia da Interação Patógeno-Hospedeiro (PPGBIO-Interação), Instituto Leônidas e Maria Deane (ILMD/Fiocruz-Amazônia), Manaus, AM, Brazil; 4 Programa de Pós-graduação em Imunologia Básica e Aplicada (PPGIBA), Universidade Federal do Amazonas (UFAM), Manaus, AM, Brazil; 5 Universidade Nilton Lins, Manaus, AM, Brazil; 6 Faculdade Estácio do Amazonas, Manaus, AM, Brazil; 7 Universidade Federal do Amazonas (UFAM), Manaus, AM, Brazil

**Keywords:** Gene libraries, phage display, sequence diversity, target affinity, biotechnological applications

## Abstract

The development of the phage display technique has brought practicality and speed when selecting high-affinity molecules. It is used to obtain single-chain variable fragments (scFvs) and has revolutionized several branches of research and industry. These are developed from gene libraries that differ in their construction strategies, which causes a diversity of sequences, specificity and binding strength of the projected molecule to its antigen. In this review, we present the recent studies that demonstrate methods and approaches using immune, naïve, synthetic and semi-synthetic libraries to construct and select scFvs. Subsequently, the characteristics of these libraries, the functionality of the scFvs and the cost-benefits of production will be discussed. In addition, we highlight the methodological trends and challenges to be overcome in order to optimize the production and application of these antibody fragments.

## Introduction

The researcher George Pieczenik Smith was the first to report in a practical way the use of the phage display technique in 1985, which caused positive impacts in the areas of immunology and molecular biology [1]. It is a method for manipulating the DNA of a bacteriophage to build phage libraries that are capable of encoding molecules to express them in the viral capsid [2].

Currently, this technique is the main molecular tool used to select the single-chain variable fragment (scFv). The scFv is a functional antigen-binding domain containing approximately 30 kDa, formed by the light-chain variable region (V_L_) and the heavy-chain variable region (V_H_) and joined by a peptide linker [3]. This antibody format can be rapidly constructed, expressed in different hosts and, due to its reduced size, greater stability and high specificity, has advantages in therapeutic applications and diagnostic tests [4, 5].

Previous studies have reported and evaluated the development and selection of scFvs directed to numerous targets [6–10], which were selected from different gene libraries that differ in origin, construction methods, size and diversity [11]. Constructed libraries can be immune, naïve, synthetic and semi-synthetic in nature [12]. Each approach has its advantages and limitations and, depending on the nature and subsequent use of the ligand analyzed during selection, is suitable for the most varied purposes [13]. Immune libraries require prior immunization of human or animal models with specific antigens, which results in a high affinity for the isolated ligands [14].

Naïve libraries, on the other hand, do not follow classical immunization, since they use a gene repertoire that is usually derived from B cells of donor patients who have not been immunized. However, these are capable of generating molecules with different specificities [15]. Synthetic and semi-synthetic libraries also do not require immunization and can be prepared using bioinformatics analysis to improve the affinity of the molecule, employing random combinatorial mutations in regions responsible for antigen binding [16]. Finally, from any of these possible gene repertoires, the construction of phage libraries, screening and selection of scFv that binds strongly and specifically to its target is performed [17].

Decades after of the emergence of phage display, many advances in this scientific field have already been achieved and are duly documented in a vast extension of studies. However, most reviews on the production of scFvs focus on presenting general aspects of the technique and its applications [18, 19]. Thus, there are no recent reports that describe in a compiled form the diversity, complexity and applicability of the gene libraries used in the process.

The main focus of this review is to provide an up-to-date understanding of the latest research involving scFv gene libraries, in addition to offering guidance to researchers in the field regarding the choice of the best strategy for obtaining scFvs that meet medical and industrial demands. Thus, this review presents the different categories of scFv libraries already developed in the last five years, as well as the origin of the gene repertoire, library size, selection methods, molecular strategies, quality of the selected scFvs, directed targets and the costs versus benefits of the production.

## Overcoming Limitations in Recombinant Antibody Generation Using Phage Display

At the end of the nineteenth century, it was found that serum from convalescent human and equine individuals from a bacterial infection could be used to treat this disease, both in humans and animals [20]. However, prolonged use of this therapy caused adverse reactions in patients, probably due to the presence of unknown proteins [21]. In 1960, the structure of antibodies was discovered as scientists sought a better understanding of the mechanisms behind serotherapy [22]. Advances in genetic engineering contributed to improvements in the process of obtaining and purifying antibodies, culminating in the emergence of monoclonal antibodies (mAbs) in 1970 [23]. These mAbs were obtained by hybridoma technology; however, the mAbs produced in murine models when used in human patients were not well tolerated for long periods [24].

The demand for more tolerable options from the clinical and therapeutic point of view fostered several studies with bacteriophages in the 1980 [25]. Bacteriophages are viruses found in nature that have a natural tropism to infect bacterial cells [26]. All the knowledge acquired about bacteriophages helped George Smith to develop the phage display technique when he conducted experiments at the University of Missouri in 1985, research that won him the Nobel Prize in 2018 [26].

**Fig. 1:**
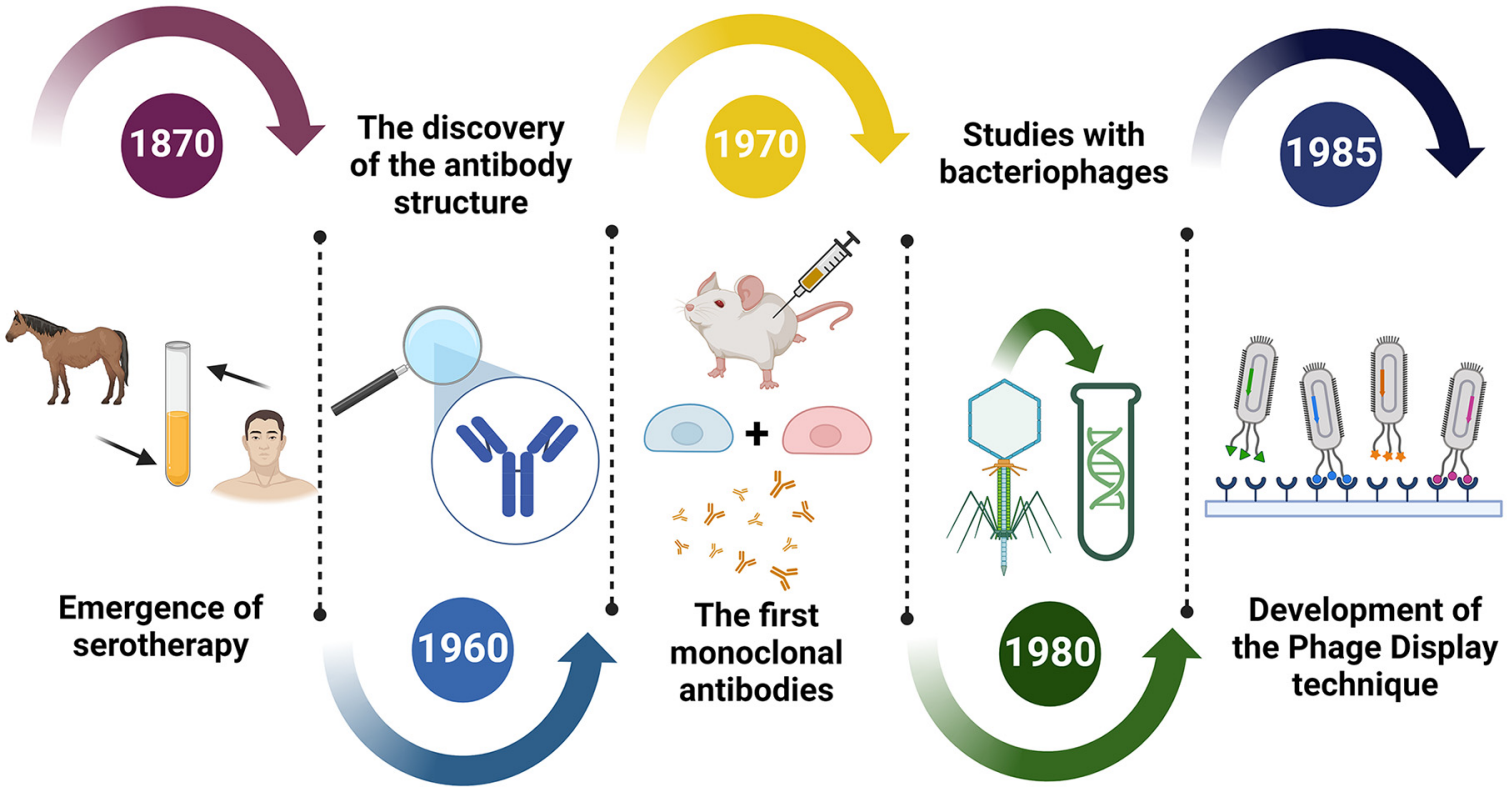
Chronology of the main advances from the discovery of the effects of serotherapy to the selection of the first recombinant molecules in phages.

In his study, Smith observed that it was possible to insert exogenous DNA next to gene III that encodes a phage surface coat protein, creating a fusion protein expressed in the viral capsid in an accessible and functional way, without compromising the infective activity of the phage [1]. Complementary studies have proven the feasibility of isolating genes of interest from random libraries [27, 28]. Below is a timeline showing the technological advances from the discovery of serotherapy to the realization of the first selections of biotechnological molecules using phage display (Fig. 1).

Compared to other methodologies used to obtain therapeutic antibodies, the main advantages of phage display include the specificity of selected clones and the diversity and size of the library that can be generated [29]. The libraries comprise millions of gene sequences, with each phage particle carrying distinct sequences [5].

All the selection is performed in vitro, which promotes the rapid isolation of binding phages in various laboratory environments, thus expanding their usefulness in emergency situations [30]. Many recent studies have described the isolation of scFvs quickly and efficiently against different targets, including in the fight against the SARS-COV-2 virus, the etiological agent responsible for causing the COVID-19 pandemic [31-34].

Although scFv selection occurs in vitro, there are increasing reports of studies that have performed in vivo selections. In these cases, the phage library is injected directly into an animal model, such as mice or rabbits, and circulating phages bind directly to their target in tissues and organs [35, 36]. Phage libraries used in phage display can be stored and preserved for long periods without compromising the capacity for infection, replication, and display of the gene of interest [37]. This high viability allows their reuse and exposure to different targets aiming at the continuous discovery of new molecular interactions [38].

Depending on the chosen molecular strategy, it is possible to join the desired gene to different viral coat proteins (pIII, pVI, pVII pVIII, pIX), which can also have their structure modified to optimize surface display [39-41]. Other elements of the technique are the vectors derived from bacteriophages belonging to the genus *Inovirus* (T4, T7, λ, M13), which do not pose a risk to humans, are easy to handle and act as appropriate vehicles to insert and display genes [40, 42].

The practicality of the technique has resulted in a considerable number of reviews that have sought to contextualize the general aspects and synthesize knowledge about the improvements that have been achieved [43-46]. The original articles present discoveries of new drugs and therapies [47-49]. Several studies have used bioinformatics strategies to identify and perform specific adjustments in the complementarity determining regions (CDRs) of scFvs involved in the interaction with the antigen [50-53], as well as report combinations of the selection technique with other technologies.

The combination of phage display with hybridoma technology is reported to improve the cloning and expression of the scFv using the genetic information of mammalian germ cell lines [54, 55]. The combination of these two approaches combined the speed and economy of phage display to complement hybridoma technology, thus optimizing time and resources [56].

Dong *et al*. [57] presented the combination of phage display with genome editing mediated by the CRISPR (clustered regulatory interspaced short palindromic repeats) technique. The objective was to generate recombinant phages capable of expressing short peptides and complete proteins in their capsid, thereby demonstrating new ways for the manipulation of the viral genome in order to generate bioactives.

Advances in tracking clones of interest have also been achieved. One example is the use of next generation sequencing (NGS) technology, which has been adopted to increase screening and thus identify a larger number of clones from an analyzed library. In the NGS analysis, additional numbers of screened clones are observed when compared to the conventional method, which is performed using colony polymerase chain reaction (PCR) [58]. The integration of phage display with NGS is currently considered a state-of-the-art method, and is indicated for analyzing a substantial amount of gene sequences quickly and effectively [53]. NGS analysis is routinely applied to assess the quality of a library by analyzing changes in heavy chain germline usage and assessing CDR diversity, particularly the composition and length distribution of CDR-H3 [59].

In the work of Krohn *et al*. [60], NGS sequencing was performed at multiple time points to verify the quality of phage libraries, monitor the selection process, and genetically characterize the antibodies. After two rounds of selection, a clear shift in the distribution of CDR-H3 lengths was observed. Furthermore, the analyses revealed that 26.204 V_H_ clonotypes were present with a 47-fold reduced effective number of species (95 clonotypes), and 0.82 x 10^6^ CDR-H3 sequences were composed of 18.926 unique CDR-H3 sequences. Ultimately, the NGS analyses successfully identified two novel antibodies, CD38 and ICAM-1, which are potential candidates for the treatment of plasma cell disorders.

Another important role of NGS in antibody discovery occurs during the methodological process of V_H_ / V_L_ pairing of scFv, which allows the creation of libraries of high diversity and specificity [61]. Choe *et al*. [62] generated and characterized a large combinatorial library of scFvs human origin based on NGS. For this, several reads and data filtering were performed to eliminate sequencing artifacts. Reads of 1.9 × 10^6^ and 2.7 × 10^6^ complete sequences of V_H_ and V_L_ domains, were obtained, respectively. In the analyzed library, approximately 94% were related to the V_H_ domain and approximately 91% were related to the V_L_ domain. These sequences were unique and, according to the NGS data, had greater diversity than the germline sequences, which validated their quality.

In addition, we emphasize that the success of the use of phage display is mainly due to its simplicity, high efficiency, in vitro nature, speed and low cost, which categorizes it as a powerful tool for selecting specific ligands. The evolution of the technique is notorious and new improvements are constantly being made, which makes this technology gain more and more prominence and it contributes significantly to the diagnosis and therapy [63].

## The Construction and Expression of scFv

**Fig. 2:**
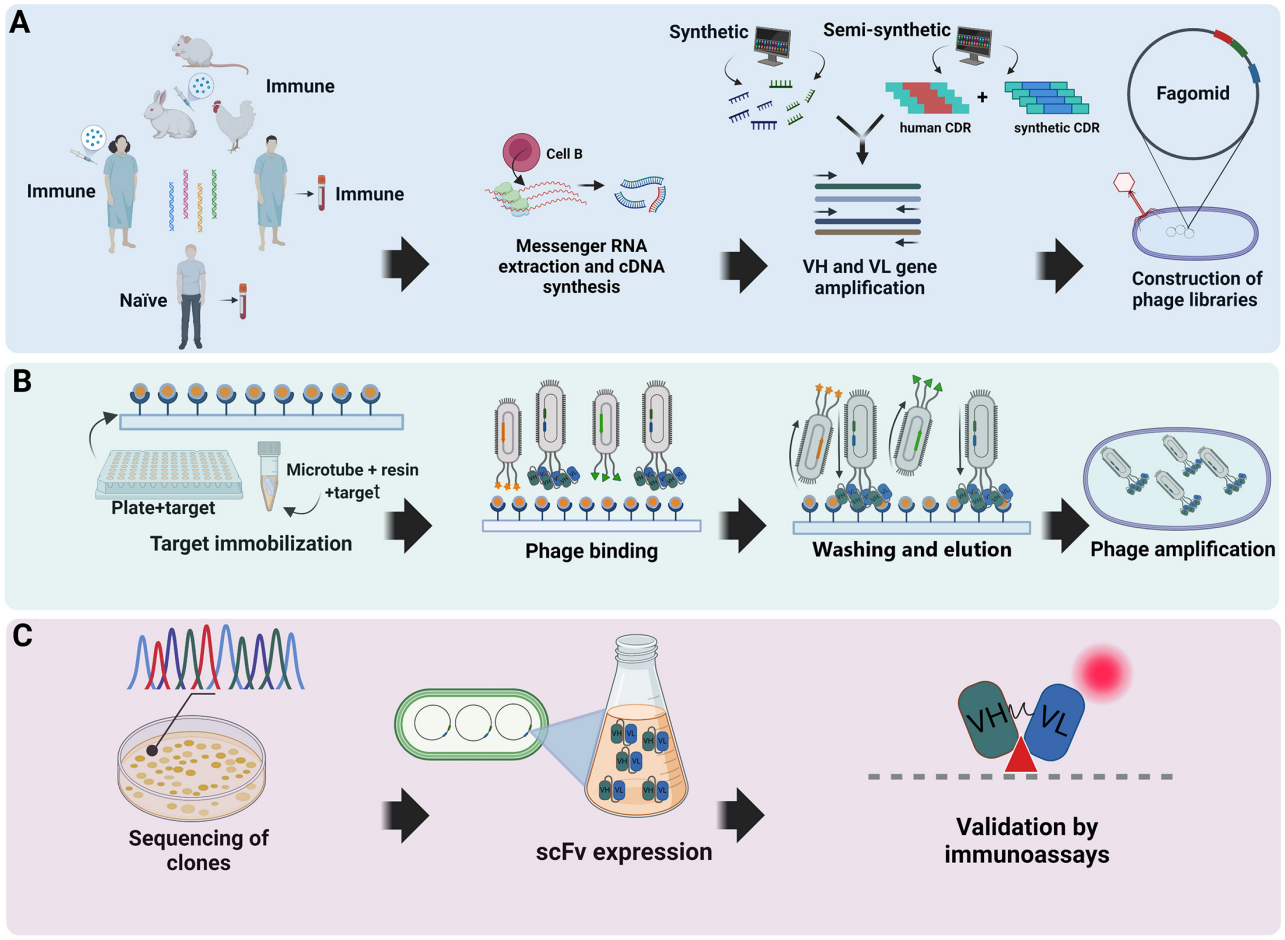
(A) Library construction steps (B) Screening, selection and elution of binding phages (C) scFv expression and functional evaluation against its target.

In general, according to the origin of the gene sequence, scFvs can be constructed and selected from immune, naïve, synthetic or semi-synthetic libraries. When the source of the antibody is of an immune or naïve nature, the construction and selection of the scFvs should include the following steps (Fig. 2A): first, mRNA is extracted from the B cells of peripheral blood of human donors, whether healthy or not, or from the blood and lymphoid organs in the case of animals [5]. Then, cDNA synthesis occurs via reverse transcriptase (RT) reactions for amplification of the variable light (V_L_) and variable heavy (V_H_) chain segments. It is possible to use specific or random primer pairs that recognize defined or conserved regions, respectively, using PCR [64].

The first PCR reaction aims to separately amplify the V_H_ and V_L_ gene. While the second reaction combines the two gene repertoires, which are assembled containing a peptide ligand, usually composed of small and hydrophilic residues of glycine (Gly) and serine (Ser) [65]. PCR is a critical step that directly interferes with cloning, and many studies present strategies for optimizing the steps and reactions to obtain a rapid construction and isolation of scFv [66-68].

During the process of building synthetic and semi-synthetic libraries, neither immunization nor manipulation of biological samples is necessary, since the entire design of the libraries is predicted through bioinformatics analyses. Synthetic libraries are chemically synthesized using oligonucleotides and semi-synthetic libraries are generated by joining synthetic regions with natural regions. These steps are performed to increase the diversity of the CDRs in the assembly process of the V_H_ and V_L_ genes (Fig. 2A).

After assembling the scFv gene from a natural or artificial immune gene source, the next step is to clone it fused to a phage capsid protein gene. For the isolation of high affinity scFvs, fusion to the pIII gene is recommended, as it results in a more controlled and precise presentation [4]. Among the available vector options, the pcomb3 family of vectors are preferable for the generation and selection of antibody fragment libraries.

A competent bacterium such as *E. coli* strain XL1-Blue is electrotransformed with the phagemid containing the scFv gene [69, 70]. Transformants are infected with helper phages such as VCSM13 [64]. After the helper phage infects the host bacterium, a complex process of assembling mature viral particles that are released into the external environment to obtain the scFv phage libraries begins [5]. The phage libraries produced are subjected to a screening and selection (Fig. 2B) by means of affinity maturation to their target after cycles of directed evolution performed via biopanning [71]. Biopanning selects phages with high affinity and binding strength to their target in plates (ELISA Phage) or microtubes containing resin and immobilized antigen [50]. The enrichment of these phage populations occurs via infection of the host bacterium, starting a new round of amplification, selection and elution [72].

Candidates identified in the first round of selection may present low affinity due to some biases such as the propagation of transforming bacteria that received the vector with the scFv gene of interest, the reduced efficiency of infection and replication of phages in the host bacteria, in addition to competition of positive clones with large numbers of mutant clones with weak affinity to the target [73, 74]. To identify high-affinity scFvs, it is recommended to perform a gradual increase in the number of screening rounds, usually, two to four rounds are necessary. This process progressively removes non-binding phages and enriches the population of phages with a higher binding strength [56].

Other parameters that affect the efficiency of biopanning screening include the concentration of immobilized antigen, the concentration of the non-ionic detergent used in the washes, and the composition of the blocking buffer [75]. Such factors require careful methodological balance, as low-stringency screening may allow retention of false positives, while high-stringency screening may result in the removal of positive clones.

The success of the bioprospection depends on the quality and format of the antigen presentation, which is crucial in the isolation of scFvs, and requires the establishment of different biopanning strategies [38, 76]. A recent study by Kamstrup *et al*. [76] focused on reducing the emergence of false positive clones, *i.e.*, clones that arise as a result of binding to one of the components of the selection system other than the target.

At the end of the selection rounds, although the selected molecules are not monoclonal in origin, they will have high affinity and binding strength comparable to or superior to conventional mAbs [56]. The final steps consist of sequencing (Fig. 2C) of the positive clone populations using two main methodologies: Sanger-type sequencing [77] or next-generation sequencing (NGS) [71]. Both approaches offer a direct analysis of clones prior to heterologous scFv expression.

The genes of the clones selected after sequencing can be expressed in different expression systems [72, 78, 79]. In addition, due to its physicochemical characteristics, the scFv is usually produced in large quantities [80]. Validation of the produced molecule can be performed using immunoassays (Fig. 2C) to ensure its complete functionality and use against its target [81, 82]. The use of immunoassays can be applied in the early stages of selection, functioning as a screening strategy for a pre-evaluation of phages fused with the scFv of interest [83, 84]. There are a variety of screening methods that can be used, such as ELISA assays, Western blot and flow cytometry, among others [85].

As an example, a recent study demonstrated that the use of immunoassays was efficient for selecting clones presented by phages against the VP0 protein of human parechovirus 1 (PeVs), an infectious agent associated with several diseases that can affect the gastrointestinal, respiratory and nervous system [86]. In the same study, the sandwich enzyme-linked immunosorbent assay (ELISA), flow cytometry and immunofluorescence were subsequently used to evaluate the binding of the purified scFv molecule to ensure its binding in the native virus. In the study by Zhang *et al*. [87], na scFv against the Bt toxin Cry1Ab from *Bacillus thuringiensis* was selected and characterized using ELISA. The scFv that presented the highest binding capacity was expressed and, after purification, the molecule was used to develop a new sandwich ELISA method that demonstrated promising results for the detection of the Cry1Ab toxin.

In addition to using ELISA for a preselection of clones, Mansour *et al*. [88] used flow cytometry to select the best candidate capable of binding to EGFRvIII, a cancer-specific receptor that can be found in ovarian, breast and glioblastoma cancers. Methods based on flow cytometry, such as FACS (Fluorescence-Activated Cell Sorting), stand out for their high throughput in the triage of libraries of recombinant antibodies, making it possible to identify and isolate recombinant antibodies in a rapid and targeted manner [89]. The use of FACS has a different advantage than depending only on real-time quantitative multiparameter analysis of individual cells, allowing the resolution of a single cell during selection [90]. This is reflected in the more precise and controlled manipulation of a population of phage clones and contributes to a greater enrichment of these populations before sequencing [91].

## scFv Production Methods Using Different Gene Sources

Table 1 presents a detailed compilation of the different types of libraries built and used in the last five years for the production of scFvs using phage display. The libraries are categorized according to their gene origin, size, molecular strategies, target and yield of scFv production.

## The Production of scFvs Using Immune Libraries

The in vivo immune response can be replicated in vitro through the construction of immune libraries [115]. Immune libraries use the diversity of genes from convalescent patients or immunized donors [116-118]. This strategy has the advantage of in vivo antibody maturation to ensure greater specificity and avidity through the combination of multiple V_H_ and V_L_ variable regions. In the process of maturation, antibody clones undergo somatic hypermutation, selection and clonal expansion, and reach large quantities, which increases the likelihood of the enrichment of high-affinity clones [119]. This causes the gene repertoire to be directed to the antigen used in the immunizations [120].

These libraries use detailed and well-described immunization protocols, which permits their experimental reproducibility. However, although there is great robustness of the method, there are reports of the construction of immune libraries that presented a lower diversity compared to other types of libraries. Rahumatullah *et al*. [121] reported the successful identification of an scFv against the parasite *Strongyloides stercoralis* from an immune library that presented a large amplitude of antibody gene diversification with the potential to be applied to different related infections.

It is worth noting that the immune system is constantly evolving depending on the health status of the host. Thus, immune libraries can offer more than just specific antibodies, but also antibodies derived from memory B cells [122]. This fact implies that there may be rapid responses to antigens already exposed to the body, making the immune response even more effective. Due to the ethical issues related to the immunization of humans and difficulties in obtaining biological samples, different animal models of experimentation, such as chicken [123], rabbit [124], mouse [125], and horse [104], among others, can also be explored regarding their immune response and specificity.

## Birds

When compared to mammalian antibodies, avian antibodies, especially those from chickens, have attributes that confer greater experimental versatility in development and applications in immunodiagnostic and immunotherapeutic assays, as well as biophysical, biochemical and bioethical advantages [126]. This is structurally different due to the presence of an additional constant heavy domain, the absence of a region of genuine hinge, and the different composition of oligosaccharides in its lateral chain [127]. At the genetic level, birds possess a single functional V gene to encode the variable region of heavy chains (V_H_, V_H_3 family) and light chains (V_L_, only light chains of type λ) [128]. However, to introduce structural variability, we use alternative mechanisms such as somatic hypermutation and alteration of amino acids in the structural regions (FWRs), responsible for the conformation of the V_H_ and V_L_ chains [129]. The CDR3 region of birds is longer, which can be highly beneficial in the construction of scFvs because longer loops are more stable and have greater sequence diversity and binding capacity to a greater diversity of antigens in comparison to other species [130].

Due to these characteristics, the IgY antibody is constantly being explored to be converted into scFv fragments, becoming a smaller molecule with high specificity [131]. In the work of Wang *et al*. [92] a specific scFv was identified against N-glycolylneuraminic acid (Neu5Gc), a type of sialic acid that when ingested can cause serious diseases such as cancer. The anti-Neu5Gc scFv of chicken origin was used in the ELISA as a detection probe and was shown to be a potential input in immunological assays for the detection of Neu5Gc in foods and tumor tissues. In the study by Mwale *et al*. [93], a laying hen was immunized with *Coxsackievirus* A16 (CA16), a virus that can cause meningitis and other infections in children. After the construction of the scFv libraries, five screening cycles were performed. The selected scFv was able to neutralize the CA16 virus and react with the viral protein lysate in the ELISA.

In another study, this time by Schoenenwald *et al*. [132], domain III of the Usutu virus envelope protein was used for generation of scFvs. The affinity, performance and neutralization capacity of the molecule was analyzed. Four scFvs showed good performance in binding tests. Although they have not neutralized the Usutu virus, they can still be candidates in diagnostic tests due to their high specificity that was observed. Lee *et al*. [95] developed an accessible therapeutic strategy for snakebites by building two libraries of scFvs against a protein from the venom of *Trimeresurus stejnegeri*, known as the “bamboo viper”. The generated libraries contained 4 × 10^7^ and 6.8 × 10^7^ clones, respectively. In in vitro assays, the scFvs produced showed reactivity against the target protein; while, in vivo tests, partial protection was observed in mice.

Dabiri *et al*. [96] developed an scFv against the extracellular domain (PTPRN) present in beta cells of the pancreas. Ostriches were used as animal models in this study. ScFv was expressed in soluble form and was characterized by different immunoassays. In the end, the scFv demonstrated a strong link to its target, this being the first study reporting the use of ostriches as a host to generate anti-PTPRN scFv candidates for the treatment of diabetic patients.

## Mice

The micés immune system is capable of generating rapid and robust responses against a wide variety of antigens, being considered an effective source for generating antibodies with high specificity and affinity [125]. The general structure and architecture of mouse IgG is similar to human’s, however, small differences in the CDR regions may result in immunogenicity, which may compromise the therapeutic applicability of these antibodies in humans [133]. To reduce immunogenicity, these antibodies can undergo a humanization process. In general terms, humanization comprises different strategies to reduce the immunogenicity of non-human antibodies without losing or drastically altering their functional properties [134]. The main humanization methods are chimerization and grafting of complementarity-determining regions (CDRs).

Chimerization generates antibodies in which the constant regions of murine origin are replaced by human constant regions, and then the residues of the murine variable framework region, except the CDRs, are also replaced by their human equivalents [135]. CDR grafting is the procedure that involves transferring CDRs from a murine “parent” antibody to the framework of a human antibody [136]. Although these humanization methods reduce immunogenicity, it is common to observe a significant drop in antibody affinity for the antigen [134, 137]. Thus, the use of scFvs is a promising alternative since they have desirable therapeutic characteristics and their simple structure allows the exploration of different production and modification strategies to improve their efficacy [138]. Among mammals, mice are often used to construct libraries of scFv. The use of these animals is already well established, with well-described protocols [139]. Most antibody fragments that have been evaluated in preclinical trials originate from these libraries and there are still many molecules that remain under investigation [140].

Recently, Martviset *et al*. [97] produced and characterized three scFvs capable of recognizing distinct epitopes of cathepsin F from *Opisthorchis viverrini*. ScFvs have been characterized from the structural and functional point of view and are new candidates for diagnosing parasitic infections. Another study showing the use of scFvs against parasitic agents was conducted by Dolgikh *et al*. [99]. The authors reported the production of these fragments against the chimeric antigen NbAAC1-3 in order to inhibit the growth of the parasite *Nosema bombycis*. In this work, after the construction of the phage library, the scFvs were expressed in Sf9 insect cells and the transformant cells were infected with microsporidia spores. The expression of the *PTP2* gene, a molecular marker of *N. bombycis* parasite growth, was analyzed via quantitative reverse transcription PCR (RT-qPCR). The results of the analyses of the transcripts showed that the transformant that expressed the scFv5 fragment showed a greater inhibitory activity, with a lower number of *PTP2* transcripts.

A study by Baurand *et al*. [98] produced scFvs for imaging purposes in the detection of protein 15 (LRRC15), an important marker of fibroblasts associated with cancer. Screening using flow cytometry resulted in the selection of 28 clones. The selected scFvs were modified by the addition of a cysteine and then expressed in mammalian cells. The validation of scFvs was performed using flow cytometry, in which the candidate Cys-ScFv F4 was specific for the LRRC15 protein. In addition, the free cysteine in the C-terminal part of the molecule makes this scFv a potential biomarker for use in future imaging tests.

Seeking to overcome the limitations of conventional sequencing methods, Nannini *et al*. [53] employed the third-generation single molecule real-time sequencing (SMRT) technique. The CD160 and CD123 libraries were analyzed and, at the end of the screening rounds, a range of functional scFvs were selected with a power of sequence analysis and selection superior to commonly used strategies.

In the veterinary field, Dormeshkin *et al*. [100] reported combining the VHH-Fc fusion protein derived from a previously constructed synthetic library with an scFv antibody for the development of a novel immunoassay against bovine pregnancy-associated glycoprotein (PAG) (*Bos taurus*). Based on the results of binding between the molecules, this test prototype showed potential for use against other antigens of interest in the areas of veterinary medicine, research and industry.

## Rabbits

In the study by Xu *et al*. [103], a single-chain antibody scFv against microcystin-LR (MC-LR), a toxin that pollutes water and agricultural products, was obtained. Four selection rounds were performed, in which 18 clones were identified. Among these, the scFv (RscFv3) was successfully expressed in a bacterial host with a yield of 796.7 μg/ml. In the ELISA, the high sensitivity of the molecule (0.03 and 0.05 μg/l) was confirmed, which makes RscFv3 a strong molecular agent to assist agricultural companies in monitoring MC-LR contamination in water samples.

Rodríguez *et al*. [101] used phage display to create an scFv that detects chicken ovomucoid SR-G1, an allergen present in chicken eggs. Therefore, rabbits were immunized and the selected clones were submitted to an indirect ELISA. The detection and quantification were significant (43 and 79 ng/ml of ovomucoid) and the results were similar to the values observed in the reference assay for allergen detection in food products. Kumada *et al*. [102] evaluated a screening method for rabbit scFvs using antigen-coupled multilamellar vesicles (Ag-MLVs). In this work, four different libraries were built. Biophysical analyses revealed that rabbit scFvs were more stable when compared to mouse scFvs. The combination of isolation of scFvs from rabbits mediated by Ag-MLVs leads to new more efficient selection approaches.

## Humans

A library named GALBLA1 was built by Effer *et al*. [105] from peripheral blood mononuclear cells from seven patients with gallbladder cancer (CBG). The library presented a diversity of 6.10 × 10^10^. The clones showed high specificity against the claudin 18.2 protein, known to be overexpressed in patients with CBG. The scFvs generated appear as new candidates for the production of human mAbs to treat patients with gallbladder cancer, as well as other gastrointestinal cancers.

To combat tetanus, a disease that primarily plagues children in many developing countries, Nejad *et al*. [106] developed a fragment of an scFv antibody with a high sensitivity for the detection of tetanospasmin, also called tetanus toxin, which is produced by the bacterium *Clostridium tetani*. Four rounds of selection were required and high affinity clones against their target were identified via ELISA. The expression of the scFv was confirmed in SDS-PAGE gel and a Western blot immunoassay, with a molecular mass of 32 kDa.

A convalescent COVID-19 patient, who had been infected with SARS-CoV-2 B. 1.617.2 (Delta), provided the gene source for the construction of the immune library in the work of Mendonza-Salazar *et al*. [107]. The target antigen of this selection was the wild-type SARS-CoV-2 receptor binding domain (RBD). The strategy to evaluate the quality of the generated libraries was based on the sequencing of five clones of each library (20 clones in total). The sequencing results showed that all clones presented unique sequences with the appropriate scFv configuration. In the end, neutralization tests suggest that the convalescent patient’s repertoire offered protection against SARS-CoV-2 Wildtype, Delta and Omicron.

## The Production of scFvs from Naïve Libraries

In selection strategies in which the objective is to increase the probability of isolation of scFv against, supposedly, any antigen, naïve libraries are also an option that uses the natural repertoire of healthy donors to create a great genetic diversity with good target coverage [141]. These libraries are used to select target-specific ligands independent of donor immune status [115]. Such a feature occurs due to the fact that gene pools of non-immune immunoglobulins are “universal”, in other words, they can contain antibodies to multiple antigens, and therefore it is unnecessary to build a new library for each antigen [5]. This is desirable especially in cases where working with unstable or highly toxic antigens for immunizations occurs [141].

A striking feature of naïve libraries is that the molecules usually have low affinity compared to antibodies isolated from immune libraries; this reinforces the importance of the size of the library generated to ensure greater affinity [142]. The low affinity occurs due to the naïve repertoire not being subjected to the affinity maturation process in vivo, and therefore the production of higher affinity antibodies does not occur [120]. For this reason, when it comes to the relationship of library size with affinity towards the target, it is recommended to building naïve libraries with larger sizes is recommended, precisely to ensure success in isolating antibodies with the highest affinities, since the average size of naïve libraries is in the range of 10^9^ [142].

However, the advantages of the naïve library are the speed of its construction, making it useful in emergency situations, as they do not use experimental animals or long immunization protocols. However, they are able to produce antibodies close to human germline genes with a low risk of immunogenicity [115]. As for its applicability, the naïve library is a preferred type of library for the development of antibodies against infectious agents, such as the bacteria *Salmonella typhus* [141] and *Mycobacterium tuberculosis* [143], the rabies virus [144] and Venezuelan equine encephalitis virus (VEEV) [145], *Candida albicans* 3153A [146] and against the malaria-causing parasite *Plasmodium falciparum* [147].

## Healthy Patients

Dong *et al*. [108] used blood, bone marrow and umbilical cord blood samples from 440 healthy donors to generate a large natural human library. Initially a primary library with a diversity of 5 × 10^7^ was generated. Subsequently, a second library (1 × 10^11^) that was improved and based on the Cre-LoxP enzyme was built to screen for the recombinant PCSK9 protein. During the selection, scFv 3D2 demonstrated a higher binding strength against the target, in addition to inhibiting LDL cholesterol in in vitro tests. This antibody was even able to increase LDLR levels when combined with statin, demonstrating good results in lowering total cholesterol.

Erasmus *et al*. [148] described the construction of a library of high-quality scFvs with a size of 5 × 10^9^ using CD19+ cells purified from a single healthy donor. This strategy was able to provide sufficient diversity to select hundreds of antibodies against different targets, despite the presence of a higher level of clonal dominance when compared to libraries created from multiple donors.

In the study by Sumphanapai *et al*. [109], the proposal was to develop an scFv that would aid in the treatment of acute myeloid leukemia (AML). The naïve library (Yamo I) was used for this purpose and the scFv coding sequence was merged with C-terminal 6 x His and c-Myc tags. The selection took place after three rounds of bioprospecting. In the functional evaluation performed using flow cytometry, clone y1HL63D6 bound specifically to its target present on the cell surface. This scFv served as the basis for the generation of an antibody in the form of IgG immunoglobulin that showed remarkable therapeutic activity against the target cell HL-60.

## The Production of scFvs from Synthetic and Semi-Synthetic Libraries

An alternative to the low affinity problem can be solved by synthesizing the scFv artificially. In these cases, synthetic libraries capable of containing billions of functional antibodies are used, whose diversity is derived from the oligonucleotides used in the assembly of the library [149]. Using the sequences available in databases, changes are made in the CDRs of the antibodies obtained and simulations are made to compare them with germline CDR sequences, removing less frequent and undesirable sequences [150]. The revised CDR sequences are assembled to produce the final scFv library [150].

In contrast, the semi-synthetic libraries of scFvs combine only CDRs that were synthesized *in silico* with stable natural structures, randomly selected to generate diversity [151]. Success in designing highly functional synthetic and semi-synthetic libraries will depend on detailed knowledge of antibody function and structure orpo [152]. The use of such libraries has been increasing for the development of scFvs in cell therapy [153] and diagnostics [154].

The main advantage of the use of *in silico* methods is their ability to search for variants of the virtual library in a short time, in addition to allowing a better understanding of antibody-antigen interactions and structural analysis of this interaction through different algorithms [155, 156].

## Synthetic scFvs

In the work of Huang *et al*. [110], a Giga-sized library of synthetic scFvs, *i.e.*, containing billions of different scFv gene sequences, was constructed. The screening took into account the diversity of the CDR as a requirement for the generation of the molecule. To ensure greater stability for the scFv, six CDRs were chosen. The strategy was to generate a configuration similar to human CDRs. These reformulations were performed using bioinformatics prior to synthesis and recombination. From the phage libraries, three antibodies recognized and inhibited the activity of T-cell immunoglobulin-3 (TIM-3), revealing the potential of the library built for biomedical research.

In the work of Bai *et al*. [111], the goal was also to increase the diversity of the CDRs. First, a previously constructed library that demonstrated a low diversity in previous studies was analyzed before and after selection to verify the improvement of amplification efficiency. In this study, the authors used the machine-learning method to predict the sequences, mainly of CDR-H3. At the end of the selection, the new library had more ligands compared to the previous one, and proved to be highly functional. Another important finding of this study is that the sequences of the CDR-H3 family can probably be determinant for the amplification of scFv clones during phage display.

## Semi-Synthetic scFvs

Valadon *et al*. [113] built a library called ALTHEA Gold Libraries. The researchers used synthetic human IGHV and IGKV germline genes and combined the H3/J (H3J) regions (CDRs). Amino acids were added at points of interaction with the antigen to increase diversification. The generation of the library followed three steps, the first was used for the combination with H3J, the second selected stable antibodies and, in the third, antibody fragments were again combined with H3J from donor mononuclear cells. The scFvs were able to recognize all the seven targets tested; in addition, they proved to be resistant to temperatures of 75°C and 80°C.

Aghdam *et al*. [112] optimized the culture conditions in *E. coli* to generate large-scale functional scFv to be used later in preclinical studies. The recombinant anti-G17-Gly scFv was previously isolated from a semi-synthetic library called the Tomlinson I Library, which was based on the unique human structure, and was randomized to V_L_ (DPK9 and Jk1) and V_H_ (V3-23 and JH4b) chains against the peptide hormone G17-Gly. To improve expression, temperature and induction conditions were modified. At the end of the work, the semi-synthetic library generated functional scFvs with a high yield (17.35 mg/l).

In their study, the target of Ghaderi *et al*. [51] was the extracellular domain of the PD-1 protein, belonging to the CD28 receptor family, which is expressed by a number of human tumors. The libraries used in the study were semi-synthetic human Tomlinson I + J libraries. After four rounds of selection, the phages were evaluated for their binding specificity in an ELISA. The scFvs were expressed in their soluble form, confirmed by Western blot and, finally, a flow cytometry assay determined the binding capacity of the scFv, which exhibited specific binding to the PD-1 antigen and stimulated Jurkat T cells on the cell surface.

Jalilzadeh-Razin *et al*. [114] selected ten scFvs with high binding affinity that ranged from 0.68 to 8.0 nM, whose prediction by molecular docking revealed that eight of these scFvs were able to detect the denatured form of the CCK2R protein, a G-protein coupled receptor (GPCR) that is associated with signaling pathways of the gastric carcinogenesis process.

## Conclusion

The choice of the gene source is a crucial step for the construction of libraries, as it determines the composition of the scFv gene repertoire and influences characteristics such as diversity, specificity and functionality of the antibody that directly impacts its application. Even after decades of consolidation of the technique for the selection of scFvs using phage display, recent studies have revealed that the strategies used still adopt the use of different libraries for various purposes, which indicates that there is no absolute consensus on the best strategy to be used. However, we noticed a methodological trend regarding the use of immune libraries, despite some related disadvantages, such as their limited diversity. However, the high specificity and affinity of scFvs from these libraries are desirable characteristics that bring efficiency in the selection and relevance for its applicability. We emphasize that there are still challenges to be overcome in the construction of scFvs, which can be overcome by integration with emerging technologies such as genome editing and bioinformatics techniques aimed at increasing quality and specificity. We also highlight that this molecule has enormous potential for high scalability, and is therefore ideal to meet the demands in clinical, medical and industrial research. Finally, we hope that this study will provide a current view of the knowledge about phage gene libraries and the applications of scFvs to guide professionals in the field in the adaptive alignment of their research according to the target studied.

## Figures and Tables

**Table 1:** Different types of gene libraries built between 2019 and 2024 for the production of scFvs using phage display.

Library	Gene source	Size	Phagomide	Fusion Gene	Helper Phage	Target	Yield	Reference
Immune	Chicken	1.7 × 10^7^	pCANTAB5E	geneIII	M13KO7	N-glycolylneuraminic acid (Neu5Gc)	26.5 mg/l and 36 mg/l	[92]
Immune	Chicken	4 × 10^6^ and 5 × 10^6^	pComb3X	geneIII	VCS-M13	*Coxsackievirus* A16 (CA16)	__	[93]
Immune	Chicken	1.5 × 10^7^	pComb3XSS	geneIII	M13K07	Usutu virus	__	[94]
Immune	Chicken	2.4 × 10^7^ and 6.8 × 10^7^	pComb3X	geneIII	M13	Protein TS	__	[95]
Immune	Ostrich	2.1 × 10^8^	pSEX81	geneIII	M13KO7	Protein tyrosine phosphatase (PTPRN)	__	[96]
Immune	Mouse	1.4 × 10^12^	pSEX81	geneIII	M13KO7	Cathepsin F	__	[97]
Immune	Mouse	1.2 × 10^7^	__	__	M13K07	Human leucine (LRRC15)	1.0-4.5 mg/ml	[98]
Immune	Mouse	1 × 10^6^ and 1 × 10^8^	pHEN1	geneIII	__	CD160 and CD123	__	[53]
Immune	Mouse	1.0 × 10^7^	pSEX81	geneIII	M13 KO7ΔpIII	NbAAC1-3	__	[99]
Immune	Mouse	1 × 10^11^	pIR-DD	geneIII	M13KO7	Bovine pregnancy-associated glycoprotein (PAG)	3.2 mg/l	[100]
Immune	Rabbit	3 × 10^6^	pComb3X	geneIII	VSCM13	Ovomucoid protein	__	[101]
Immune	Rabbit	10^12^ – 10^14^	pPLFMAΔ25 0pIIIpMM	geneIII	VCSM13	IgG human policlonal (huIgG), IgA human (huIgA), CRP and NP-A and NP-B the influenza virus	__	[102]
Immune	Rabbit	3.26 × 10^9^	pIT2	geneIII	KM13	Microcystin -LR	3.98 mg/l	[103]
Immune	Horse	10 × 10^6^ and 5 × 10^7^	pCC16	geneIII	M13KO7	Native toxins BoNT/A and BoNT/B	__	[104]
Immune	Human	6.12 × 10^10^	pCDisplay3	geneIII	M13K07	Claudine 18.2	__	[105]
Immune	Human	8.7 × 10^7^	pSEX81	geneIII	M13K07	Tetanospasmin	45 μg/ml	[106]
Immune	Human	5 × 10^12^	pADL	geneIII	CM13K	Receptor-binding domain, (RBD)	__	[107]
Naïve	Human	1 × 10^11^	pDF	geneIII	M13K07	Subtilisin/kexin convertase type 9 (PCSK9)	50-200 mg/l	[108]
Naïve	Human	5 × 10^6^	pMod1	geneIII	M13K07	Cell HL-60	120 mg/l	[109]
Synthetic	Human	2.5 × 10^10^	pCANTAB 5E	geneIII	M13KO7	Immunoglobulin-3 cells T (TIM-3)	10 mg/l	[110]
Synthetic	Human	1.3 × 10^10^	pComb3X	geneIII	VCSM13	hNinj1	32 mg/l	[111]
Semi-synthetic	Human	1.47 × 10^8^	pIT2	geneIII	M13KO7	Peptide hormone G17-Gly	17.35 mg/l	[112]
Semi-synthetic	Human	1.2 × 10^12^	pIT2	geneIII	M13KO7	Protein PD-1	__	[51]
Semi-synthetic	Human	2 × 10^9^	pADL-23c	__	M13KO7	TNFα, HSA, HEL	50-100 mg/l	[113]
Semi-synthetic	Human	6.8 × 10^11^	pIT2	geneIII	KM13	CCK2R	0.3-1.34 mg/l	[114]

Note: ( - ) Not informed.
